# Species-specific glucose mineralization in artificial soil: insights from *Bacillus subtilis* and *Streptomyces cinnamoneus* mono- and co-cultures

**DOI:** 10.1128/spectrum.00605-26

**Published:** 2026-06-10

**Authors:** Kanade Fujiwara, Tomoyuki Makino, Toru Hamamoto

**Affiliations:** 1 Graduate School of Agricultural Science, Tohoku University, Sendai, Miyagi, Japan; Connecticut Agricultural Experiment Station, New Haven, Connecticut, USA

**Keywords:** artificial soil, C mineralization, r/K strategist, interspecific interaction

## Abstract

Soil microbes play a critical role in carbon (C) cycling, yet assessing the direct impact of individual microbes and their interactions on C mineralization remains poorly understood due to confounding effects under fluctuated physicochemical and biological variables in natural soils. “Artificial soil systems” enable precise control in a uniform environment to directly quantify species-specific carbon cycling, yet it has not been sufficiently demonstrated whether they can isolate competitive dynamics and metabolic roles of individual microbes in C mineralization. This study used an artificial soil system to quantify glucose mineralization through a 14-day incubation experiment using *Bacillus subtilis* NBRC 101584 (*B. subtilis*) and *Streptomyces cinnamoneus* NBRC 13823 (*S. cinnamoneus*). In monoculture incubations, *B. subtilis* showed a rapid mineralization rate and high cumulative respiration, whereas *S. cinnamoneus* exhibited delayed and lower respiration. In co-culture incubation, cumulative respiration and DNA yields converged to that of the *B. subtilis* monoculture, and positively correlated with its relative abundance. In contrast, *S. cinnamoneus* markedly reduced soil pH despite lower respiration, highlighting a distinct ecological trade-off. These findings demonstrate that artificial soil systems provide the basis for directly evaluating species-specific mineralization patterns, competitive interactions, and microenvironmental modifications. This controlled approach provides an effective tool for elucidating microbial mechanisms regulating soil C dynamics.

**IMPORTANCE:**

The role of individual soil microorganisms in carbon dynamics has long been obscured by the extreme complexity of natural soil environments. This study utilized a controllable artificial soil system to directly quantify the glucose mineralization rate using *Bacillus subtilis* NBRC 101584 (*B. subtilis*) and *Streptomyces cinnamoneus* NBRC 13823 (*S. cinnamoneus*). As a result, *B. subtilis* achieved fast glucose mineralization and elevated cumulative CO_2_ production, in contrast to *S. cinnamoneu*s, which demonstrated slower mineralization and reduced overall respiration. These results highlight that artificial soil systems offer a critical and enabling framework for resolving species-specific mechanisms in soil carbon cycling.

## INTRODUCTION

One gram of soil can contain as many as 10 billion microbes (1), which play a crucial role in the soil carbon (C) cycle. Soil microbes decompose soil organic matter (SOM) to generate energy (i.e., ATP) for their metabolic activities and assimilate it into their biomass while releasing CO_2_ through respiration. Microbial products (i.e., necromass, protein, and DNA) account for 10%–80% of SOM (2), while microbial respiration accounts for more than 60% of soil CO_2_ emissions (3, 4).

Microbe-driven CO_2_ emission is often studied by attempting to link microbial ecological traits (e.g., r/K selection, Gram-positive/negative status, Genome size, GC content) to C mineralization rates (5–8). However, most studies have been conducted at the phylum level, using broad community assessments (9, 10). This approach presents several limitations. First, C mineralization rates of individual microbial species have not been directly evaluated in natural soil environments. Second, the classification of ecological traits based on community assessments or broad characteristics often yields contradictory results depending on the experimental context. K-strategists are characterized by slow growth rates and are associated with enhanced soil C sequestration, while r-strategists, characterized by rapid growth rates, dominated soil with high organic C content and thus enhanced soil respiration (5). For example, high GC content bacteria, such as Actinomycetota, are classified as K-strategists, while low GC content bacteria, such as Bacillota, are categorized as r-strategists (11). In contrast, the classification based on Gram staining categorizes both Bacillota and Actinomycetota (Gram-positive) as K-strategists (12). Meta-analytical studies show C mineralization rates correlate positively with the abundance of certain r-strategists (e.g., Bacteroidota) and negatively with certain K-strategists (e.g., Acidobacteriota), while the correlation for phyla like Bacillota and Actinomycetota often remains insignificant (9, 10). Furthermore, the complexity of natural soils is further increased by the presence of highly diverse and heterogeneous microbial communities, in which multiple populations may interact simultaneously under fluctuating environmental conditions (13). This makes it challenging to isolate the specific impact of individual species and their interactions on C dynamics in soils. Consequently, it is essential to examine individual C dynamics under in simple setting to reveal complex community functions (14). A deeper understanding of C cycling through microbial regulation requires integrating data from individual microbes and simplified microbial communities with comprehensive analyses of terrestrial microbial ecosystems (15, 16).

To address this challenge, researchers have employed simplified experimental systems. Liquid and agarose media are commonly used in microbial research to investigate interspecific interaction (17, 18). However, Del Valle (19) reported significant differences in C dynamics between liquid culture and natural soil conditions, suggesting that simplified systems might not accurately reflect microbial dynamics in natural soil environments. Sterilized soils offer an alternative approach, but the sterilization process itself can significantly alter soil chemical or physical properties (20, 21), confounding the results. Furthermore, members of some phyla (e.g., Bacillota and Pseudomonadota) are resistant to autoclaving (22). Therefore, it remains a critical gap for experimental systems that can effectively combine the simplicity of laboratory culture conditions with the physical characteristics relevant to natural soil environments.

Artificial soil systems represent another alternative approach, which allows for the controlled manipulation of physiochemical properties while maintaining a structured matrix (23–26). Previous studies using artificial soils have assessed microbial survival and mortality (20, 27). Although Guenet et al. (28) also examined C mineralization rates in monoculture, they used complex organic matter (humic acid) incorporated into the artificial soil structure as the C source. Consequently, these studies generally lacked the evaluation of potential C mineralization rates from individual species and as a functional outcome of interspecific interactions. Specifically, it has not yet been demonstrated whether these systems can be used to isolate and quantify the competitive dynamics and metabolic contributions of individual species to C cycling.

The objective of this study was, therefore, to establish and validate an artificial soil microcosm as a tool for investigating species-specific and interaction-driven C dynamics. To achieve a highly controlled assessment, we used glucose as a simple, labile C substrate. We hypothesized that (i) the artificial soil system can reflect established ecological life-strategies (r/K selection) based on C mineralization rates in monoculture and (ii) the system can isolate and visualize the mechanisms of interspecific competition as a driver of C dynamics. To test these hypotheses, we conducted a 14-day incubation experiment with two microbial species (*Bacillus subtilis* NBRC 101584 and *Streptomyces cinnamoneus* NBRC 13823), representing contrasting life strategies, in this controlled environment.

## MATERIALS AND METHODS

### Artificial soil

Artificial soil components were developed based on Ellis (20) with minor modifications (Table 1). Briefly, the matrix consisted of quartz sand (sand fraction), kaolinite, and Japanese acid clay (clay fraction). Calcium carbonate and humic acid were added to provide buffering capacity. The soil pH was adjusted to 7.2. The total C and N contents of the artificial soils were 11.5 mg g⁻¹ soil and 0.5 mg g⁻¹ soil, respectively. The artificial soils were sterilized with an autoclave (121°C, 20 min) and stored until use.

**TABLE 1:** Composition of the artificial soil

Component	Amount (g)	Proportion (%)
Quartz (sand)	27.4	68.49
Kaolinite (1:1 clay)	7.83	19.57
Japanese acid clay (1:2 clay)	3.91	9.78
Humic acid	0.78	1.96
CaCO_3_	0.08	0.20
Total	40	100

### Experimental design

Two bacterial strains were inoculated in the artificial soils: *Bacillus subtilis* NBRC 101584 (*B. subtilis*), and *Streptomyces cinnamoneus* NBRC 13823 (*S. cinnamoneus*). *Bacillus* is the genus of the phylum Bacillota, and *Streptomyces* is the genus of the phylum Actinomycetota. Both genera are among the most abundant in natural soils and have been shown to play key roles in soil organic matter decomposition (27, 29, 30). Both strains were recovered from L-dried ampoules according to the method described by manufacturer’s instructions (NBRC, Japan) and then stored at −80°C in glycerol stocks until use. Prior to inoculation into the artificial soils, each strain was cultivated and maintained in growth phase at 30°C according to manufacturer’s instructions. The bacterial cultures were then centrifuged at 2,150 × *g* for 10 min and washed with phosphate-buﬀered saline (PBS, pH 7.0) to remove medium components that could induce bacterial respiration (31, 32). Both bacterial strains were resuspended in PBS and adjusted to OD_600_ of 0.1. Additionally, DNA extraction was performed using the Mighty Prep reagent for DNA (Takara Bio, Inc., Japan), according to the manufacturer’s protocol. The concentration of extracted DNA was quantified by using the QuantiFluor dsDNA system (Promega, USA).

The microcosm consisted of 40 g (equivalent dry mass) of sterilized artificial soil component mixture placed in a sterilized 50 mL bottle (Fig. S1). The experimental design included six different microbial inoculations, and no microbial inoculation (CK) was conducted with four replicates. We inoculated the artificial soils with one of the bacterial species or prepared mixed cultures. Additionally, we prepared mixed cultures, including an equal mixture of BM and SM (SB1) with the same microbial biomass as the monocultures, and three additional mixtures (SB2, SB0.5, SB0.1) with varying proportions of *B.subtilis* (Table S1). Glucose was selected as the model substrate based on preliminary experiments where *B.subtilis* exhibited robust respiration with glucose but no detectable CO_2_ production with oxalic acid or phenol (Fig. S2). Also, glucose is known to be readily decomposed by most heterotrophic microbes and has been used in most previous research focusing on the C dynamics or C use efficiency in natural soils (8). For C mineralization measurements, a substrate solution containing glucose (0.7 mg C g^−1^ soil) and NH_4_NO_3_ (0.082 mg N g^−1^ soil) and 1× PBS buffer (0.012 mg P g^−1^ soil) was added to prevent any microbial nutrient limitation during the incubation period. The amount of applied glucose reflects the amount of C released from the plant roots into the soil in 1 week (33, 34). The CNP ratio of the substrate was equivalent to the global mean microbial biomass ratio (60:7:1) (35). The incubation experiment was then maintained by wetting 20% of the soil moisture with substrate solutions and microbial inoculums. The substrate solutions and microbial mixtures were mixed into the artificial soils using a sterilized spatula.

Microbial respiration (CO_2_ emission) measurement was performed using an alkali trap method. Each sample was placed inside a 225-mL glass jar containing 3 mL of 1 M NaOH and 10 mL of 0.01 M HCl, which was also used for keeping the water contents of the soil. We incubated at 30°C for 14 days, and 1 M NaOH was replaced new one five times (2, 4, 6, 8, and 10 days after inoculation). Exchanged 1 M NaOH was titrated with 1 M HCl. Microbial respiration was calculated by subtracting non-microbial treatment (CK) from microbial treatments. At the end of the incubation experiment, the pH of each soil sample was determined by mixing 6 g of air-dried soil with 30 mL of deionized water (pH7.55), shaking for 30 min, and measuring the pH using a pH sensor (HORIBA Scientific, Japan). Soil DNA was also extracted from the samples after a 14-day incubation period using the DNeasy PowerSoil Kit (QIAGEN, Germany). The extracted DNA was quantified using the QuantiFluor dsDNA system (Promega, USA). The DNA yield was calculated by subtracting the initial DNA amount from the final DNA amount.

### Statistical analysis

All statistical analyses were performed using R (version 3.1.1). Tukey HSD was performed to detect differences within the treatments.

## RESULTS AND DISCUSSION

**Fig 1:**
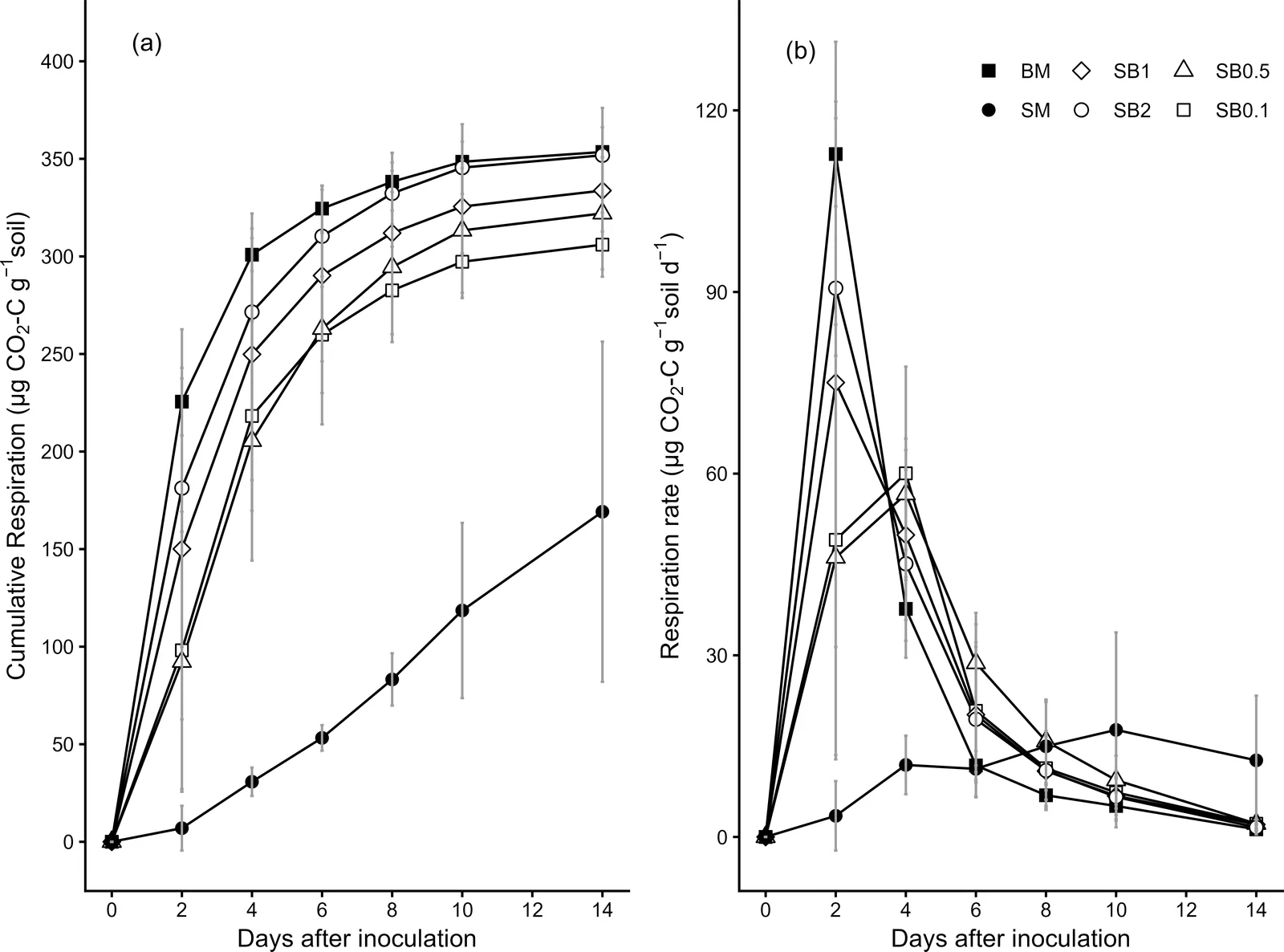
(**a**) Temporal cumulative respiration (μg CO_2_-C g^−1^ soil) and (**b**) microbial respiration rate (μg CO_2_-C g^−1^ soil D⁻^1^) over 14 days of incubation. Error bars represent the standard deviation (*n* = 4). BM: *B. subtilis*; SM: *S. cinnamoneus*; SB1, SB2, SB0.5, and SB0.1: co-culture treatments with different relative abundances of *B. subtilis* (see Table S1 for details).

As shown in Fig. 1A, cumulative microbial respiration varied significantly between monoculture treatments in the artificial soil systems. These results indicated that under a simple labile substrate condition, the two selected strains have clear functional differences consistent with temporal bacterial life strategies. While the assignment of Bacillota and Actinomycetota to r- or K-strategist categories is recognized as highly context-dependent in complex natural systems (16, 36–38), it is important to note that the primary objective of this study was not to re-validate this general r/K classification but rather to observe how the differences in life strategy are quantitatively shown as a functional status (C mineralization) under the strictly controlled conditions of a single and labile C source. The observed differences in respiration in this simple system clearly modeled the functional trade-off where each strategy utilizes the glucose substrate with different time scales (Fig. 1). Our model system showed that *S. cinnamoneus* (Actinomycetota) had significantly lower mineralization rates than *B. subtilis* (Bacillota). These differences suggest specific C dynamics driven by their contrasting life strategies. The temporal respiration patterns further supported this distinction: *B. subtilis* respiration peaked at 2 days after inoculation, whereas *S. cinnamoneus* showed a delayed respiration peak beyond 2 days after inoculation (Fig. 1b). Moreover, *B. subtilis* showed higher cumulative respiration per the amount of inoculated DNA than *S. cinnamoneus* (Fig. S3), suggesting that r-strategists have higher mineralization rates per cell mass their growth to maintain their rapid growth strategy compared to K-strategists (39). These monoculture results provide a quantitative model of how r- and K-strategists functionally express their ecological differences when utilizing a common, simple substrate within an artificial matrix.

The artificial soil system can be an effective tool for the direct and quantitative evaluation of interspecies interactions. The cumulative respiration strongly correlated with the final DNA yield across all treatments (r = 0.71, *P* < 0.005; Fig. S4), indicating that CO_2_ emission reflects microbial growth status. Notably, both cumulative respiration and DNA yields in all co-culture treatments (*B. subtilis + S. cinnamoneus*) were virtually identical to *B. subtilis* monoculture levels, and significantly higher than those of the *S. cinnamoneus* monoculture (Fig. 1 and Fig. S5). This convergence can be explained by the rapid resource acquisition of the r-strategist combined with potential inhibitory effects. In general, r-strategists uptake glucose more rapidly (27, 40, 41) and consequently have stronger resource competition than K-strategists (42). *B. subtilis* may inhibit the activity of *S. cinnamoneus* potentially through two mechanisms: forming biofilm restrict the resource availability (43, 44), and producing antibiotics (e.g., surfactin, lactonase-homologous proteins) inhibit metabolization and growth (45–49). Furthermore, our study experimentally demonstrated the regulation of community function by initial population ratio. The respiration peak was delayed with decreasing the relative abundance of *B.subtilis* (Fig. 1B). Furthermore, cumulative respirations showed a positive correlation with the relative abundance of *B.subtilis* in artificial soils (Fig. S6). These results supported previous findings that the relative abundance of r-strategists correlates positively with C mineralization rates in natural soil systems (9, 10). Despite being outcompeted in co-cultures, *S. cinnamoneus* exhibited a distinct strategy: slow growth coupled with lowering soil pH (Fig. S7). This slow growth reflects a key ecological trade-off where investment in resource acquisition is shifted to environmental modification driven by organic acid production (50–52). These findings highlight the advantage of artificial soil systems. While homogenized liquid cultures mask local changes due to rapid diffusion and buffering, the porous soil matrix restricts diffusion to preserve pH micro-gradients (53).

In this study, we acknowledge several limitations. First, only two specific strains (*B. subtilis* NBRC 101584 and *S. cinnamoneus* NBRC 13823) were evaluated, which limits the interpretation of our results due to potential strain-dependent effects and does not fully represent the vast taxonomic diversity found in natural soil environments. Second, the use of glucose alone likely favors fast-growing *B.subtilis*, whereas natural soils contain recalcitrant substrates (e.g., cellulose, lignin), allowing for niche partitioning. Third, total DNA amounts were used as an indicator of microbial biomass instead of cell numbers. However, this approach could not consider the potential inclusion of DNA from dead cells or species-specific variations (e.g., genomic DNA content, genome size), which remains a limitation. Finally, the lack of temporal population monitoring limits our understanding of community changes. However, these simplifications, by removing the background noise inherent to natural soils, enabled the direct quantification of single-species mineralization rates and the isolation of interspecific competition as a primary driver of C dynamics. This study demonstrates that the artificial soil system is a controlled tool for revealing microbial mechanisms that are difficult to isolate in natural environments. By incorporating broader species or communities in future studies, this artificial soil environment can serve as a mechanistic bridge between simplified culture experiments and complex natural soils, providing deeper insights into soil C dynamics.

### Conclusion

This study demonstrates that artificial soil systems are effective tools for elucidating microbial contributions to C dynamics. Our results showed that *B. subtilis* NBRC 101584 drove rapid glucose mineralization, whereas *S. cinnamoneus* NBRC 13823 exhibited a contrasting strategy of slow growth coupled with soil acidification. This highlights a key ecological trade-off between resource acquisition and environmental modification. Furthermore, co-culture experiments revealed that cumulative respiration converged to that of the *B. subtilis* monoculture, positively correlating with its relative abundance. These findings validate the artificial soil system as a powerful platform for quantifying single-species mineralization patterns. Future research using broader microbial communities will further bridge the gap between simplified culture experiments and complex ecosystem processes.

## SUPPLEMENTAL MATERIAL

Supplemental figures spectrum.00605-26-s0001.docx Fig. S1 to S7.

Table S1 spectrum.00605-26-s0002.xlsx Composition of each treatment.

ASM does not own the copyrights to Supplemental Material that may be linked to, or accessed through, an article. The authors have granted ASM a non-exclusive, world-wide license to publish the Supplemental Material files. Please contact the corresponding author directly for reuse.
