# Supplementary material for: Species-specific glucose mineralization in artificial soil: insights from Bacillus subtilis and Streptomyces cinnamoneus mono- and co-cultures
Source: Microbiol Spectr. 2026 Jun 10;14(7):e00605-26. doi: 10.1128/spectrum.00605-26 (PMC13340013; doi:10.1128/spectrum.00605-26)
Supplement: Supplemental figures — Fig. S1 to S7. [file spectrum.00605-26-s0001.docx]

**Title: Species-Specific Glucose Mineralization in Artificial Soil: Insights from Bacillus subtilis and Streptomyces cinnamoneus Mono- and Co-cultures**

**Authors:**

**Kanade FUJIWARA^1^, Tomoyuki MAKINO^1^, Toru HAMAMOTO^1^***

**Address:**

**^1^Graduate School of Agricultural Science, Tohoku University, 468-1 Aramaki Aoba Aoba-ku Sendai Miyagi 9808572, Japan**

***Correspondence: T Hamamoto, toru.hamamoto.d1@tohoku.ac.jp**


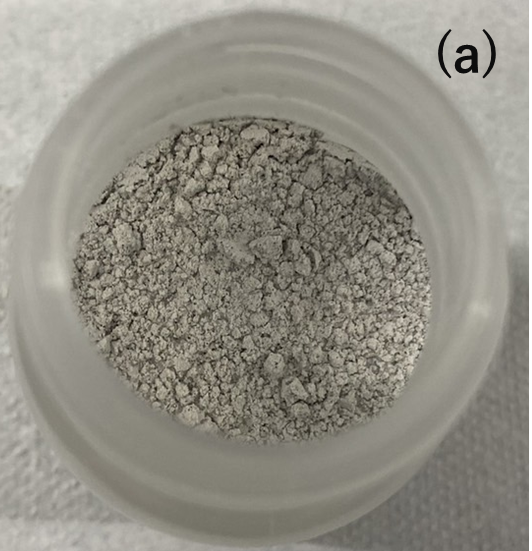
　 　
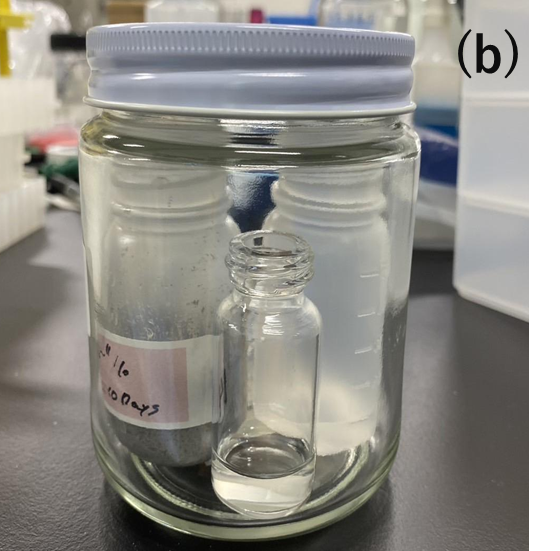


Figure S1. Photographs of (a) artificial soil mixture and (b) an alkali trap setup. The alkali trap consists of three bottles: a bottle containing the artificial soils, a glass vial with 1 M NaOH, and another bottle with 0.01 M HCl to maintain the soil moisture.

**
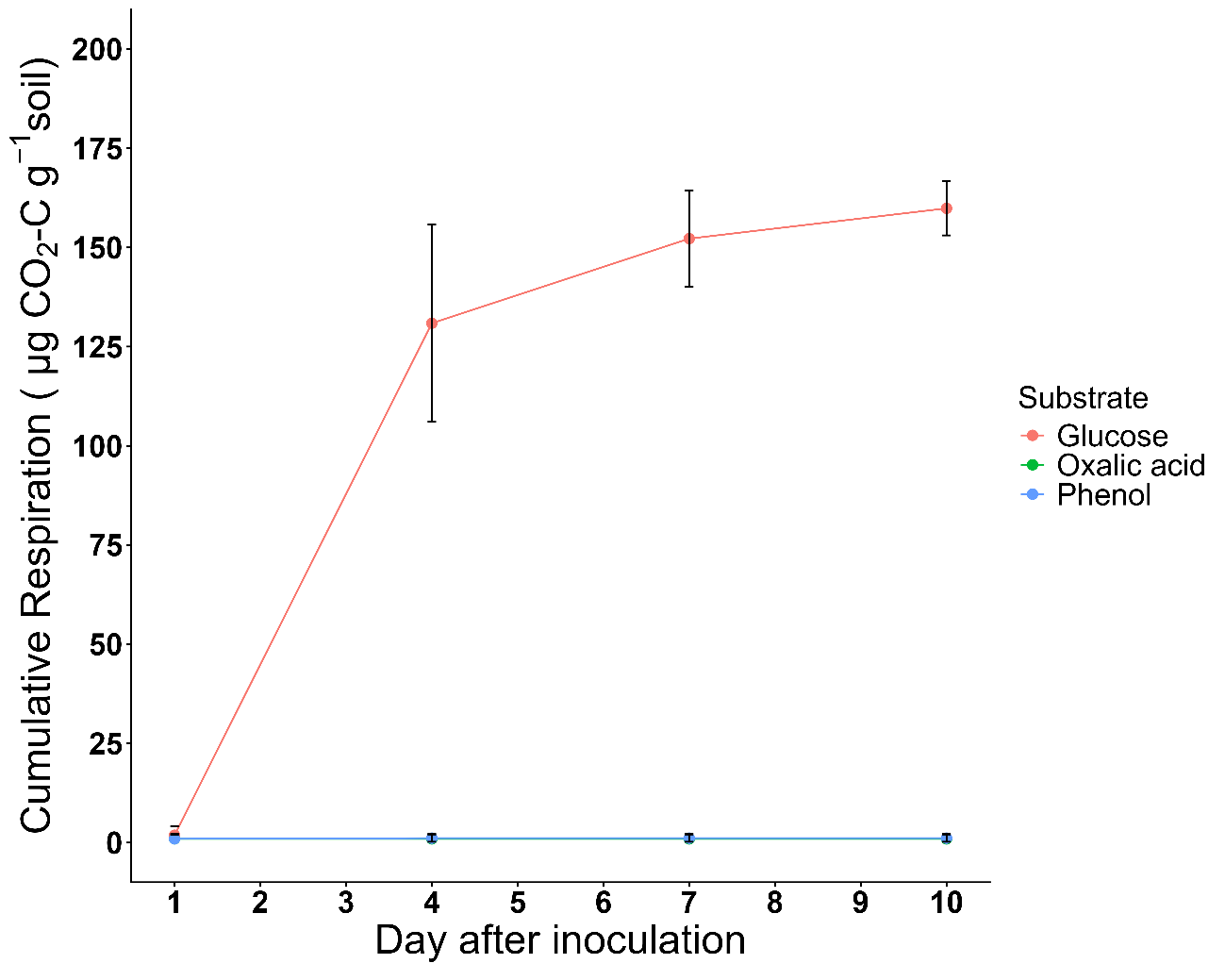
**

**Fig.S2. Cumulative respiration of *B.subtilis* (µg CO₂-C g^−1^ soil) over 10 days of**

**incubation compared to three different substrates (Glucose (Red), Oxalic**

**acid (Green) and Phenol (Blue)) (n=4).**

**
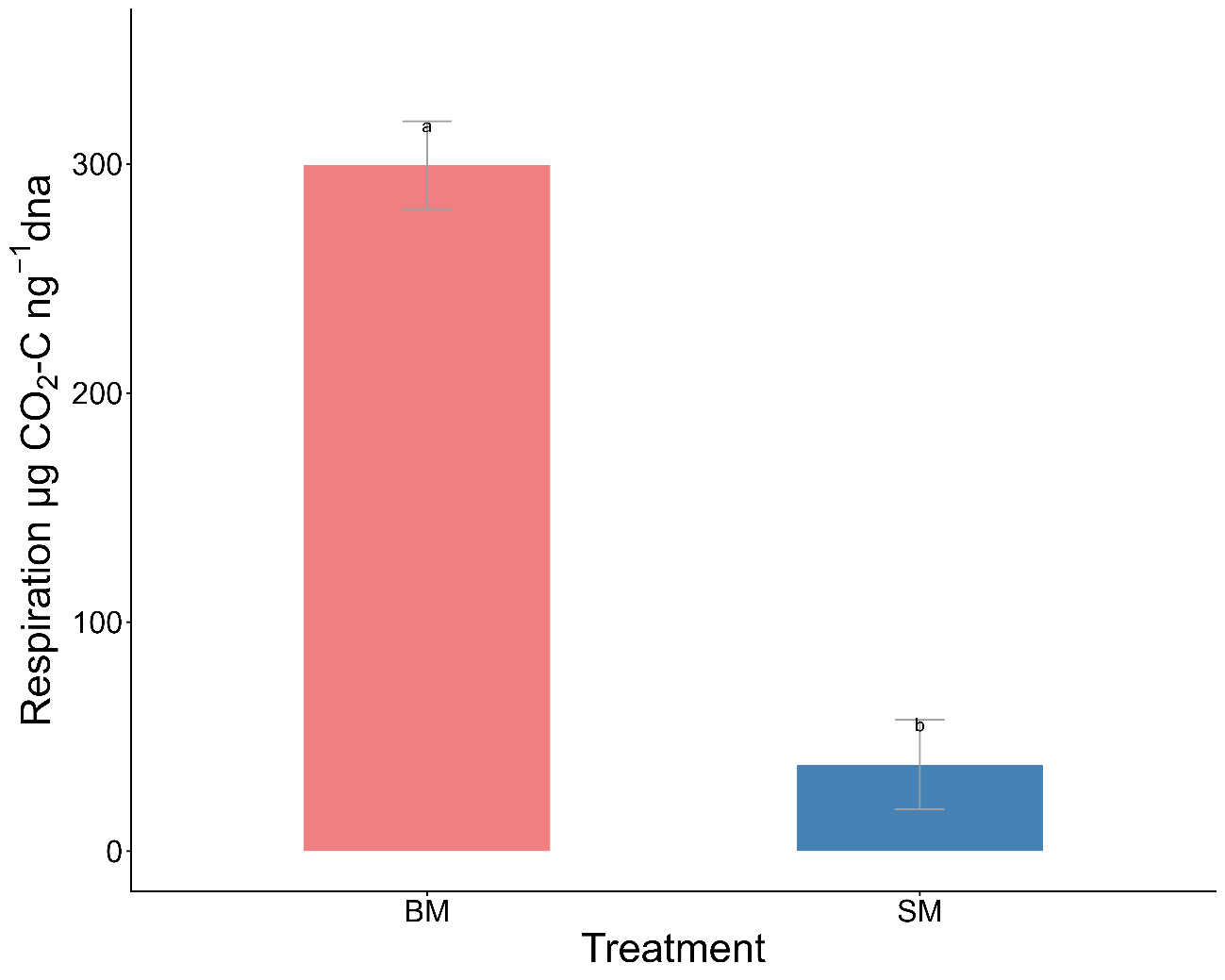
**

Figure S3. Cumulative respiration per the amount of inoculated DNA in monoculture treatments (BM: B. subtilis, SM: S. cinnamoneus). Error bars represent the standard deviation (n = 4). Different letters indicate significant differences (p < 0.05).

**
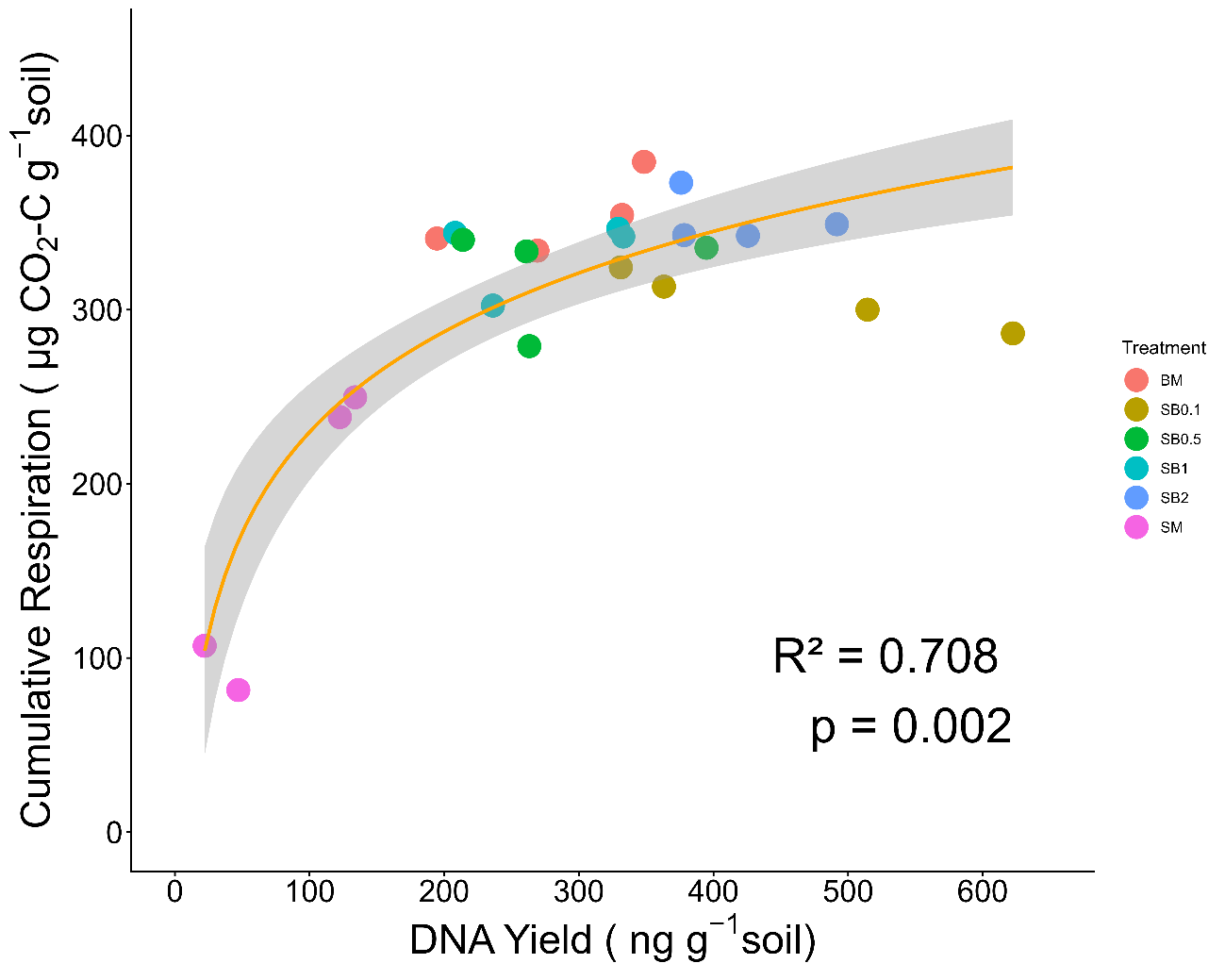
**

**Fig.S4. Correlation between DNA Yield (ng g^−1^ soil) and Cumulative respiration of B.subtilis (µg CO₂-C g^−1^ soil) (n=4). BM: B. subtilis; SM: S.cinnamoneus; SB1, SB2, SB0.5, and SB0.1: co-culture treatments with different relative abundances of B. subtilis (see Table S1 for details).**

**
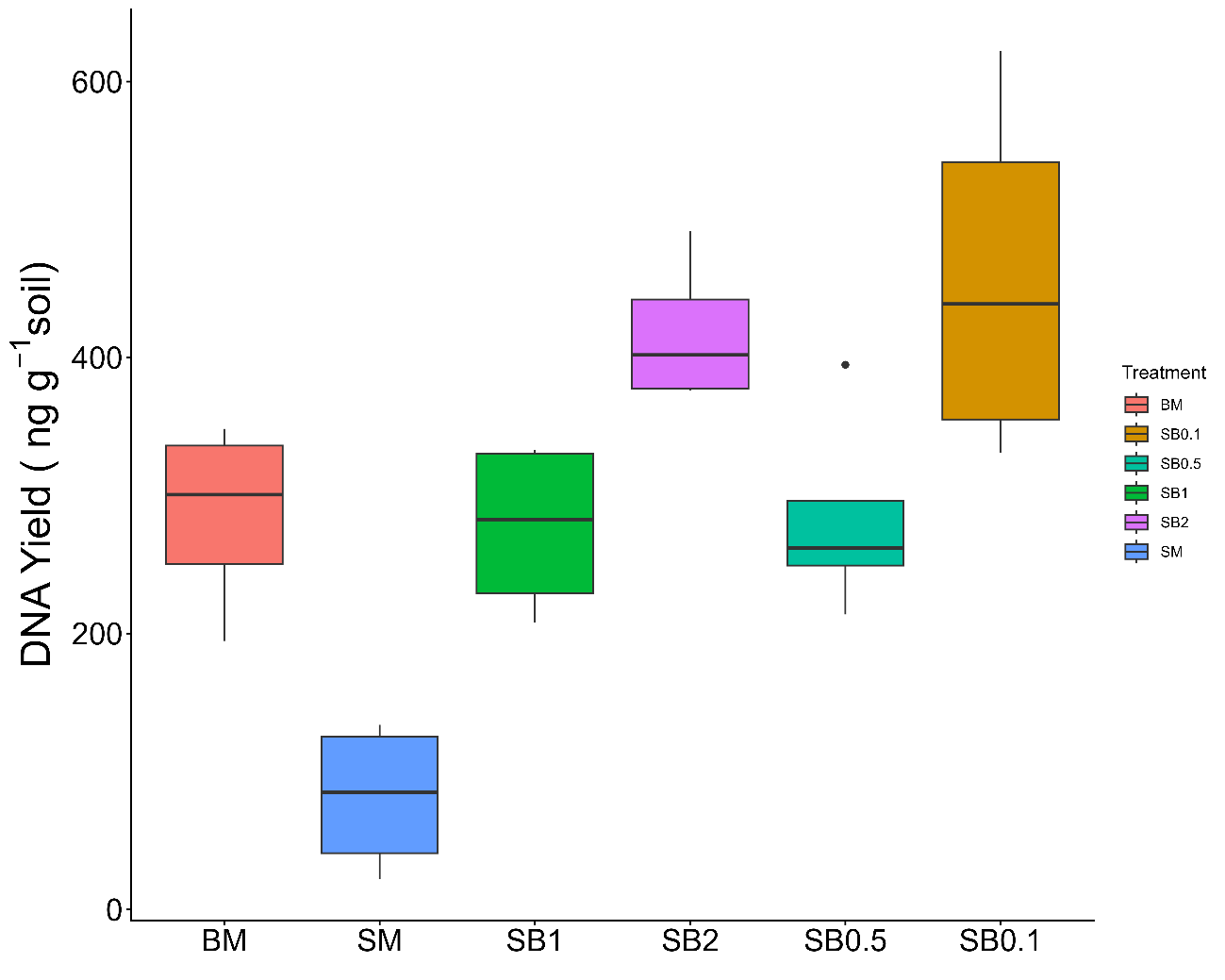
**

**Fig.S5. DNA Yield (ng g^−1^ soil) after 14 days’ incubation experiment (n=4). We extracted soil DNA using the Power Soil Pro Kit. BM: B. subtilis; SM: S.cinnamoneus; SB1, SB2, SB0.5, and SB0.1: co-culture treatments with different relative abundances of B. subtilis (see Table S1 for details).**

**
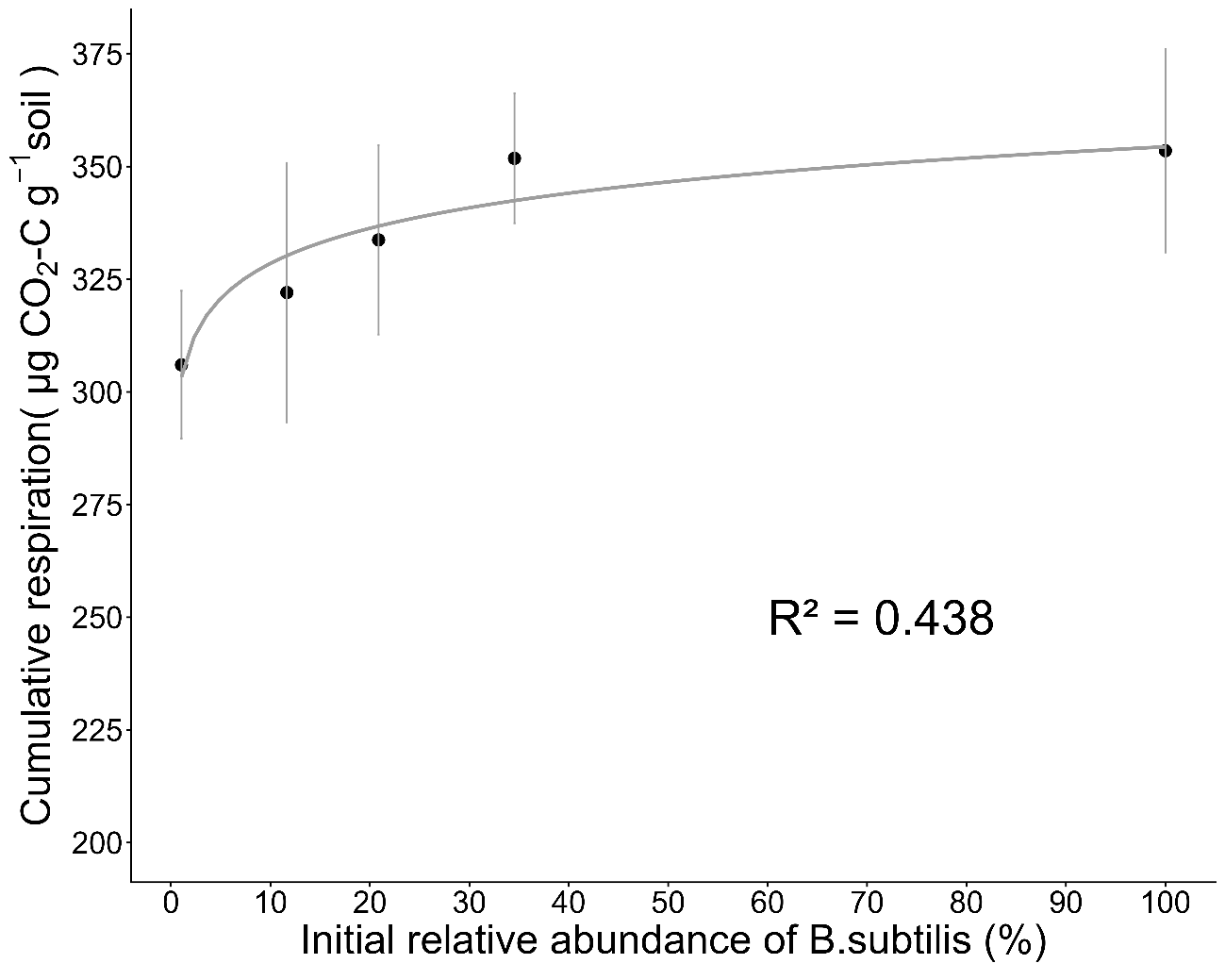
**

Figure S6. Relationship between cumulative respiration (μg CO₂-C g^−1^ soil) and the relative abundance of inoculated *B. subtilis* (%). Error bars represent the standard deviation (n = 4). The relationship was fitted using a logarithmic model (R² = 0.438).

**
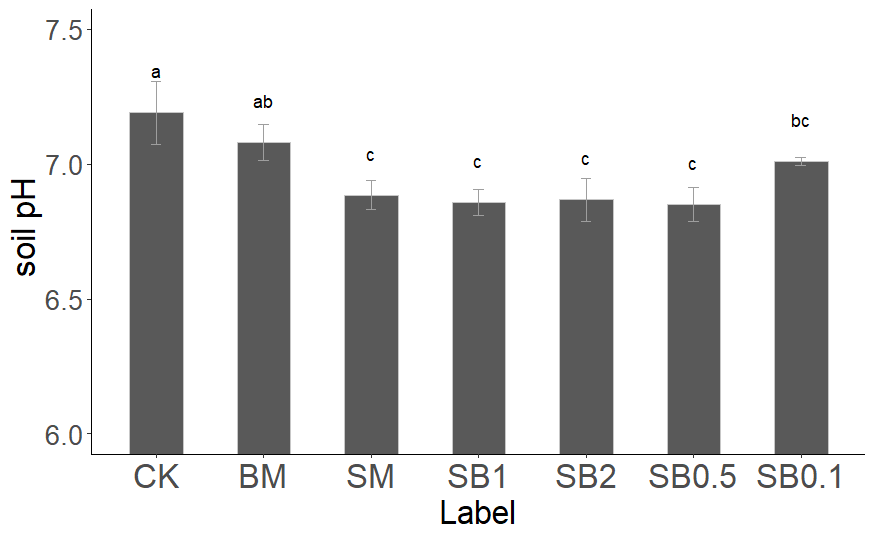
**

Figure S7. Soil pH at the end of incubation. Error bars represent the standard deviation (n = 4). Different letters indicate significant differences (p < 0.05). CK: control; BM: B. subtilis; SM: S.cinnamoneus; SB1, SB2, SB0.5, and SB0.1: co-culture treatments with different relative abundances of B. subtilis (see Table S1 for details).
